# Chronic hepatitis E virus-induced spinal cord atrophy in a patient with chronic lymphatic leukemia: a case report and interdisciplinary management proposal

**DOI:** 10.3389/fimmu.2024.1445944

**Published:** 2024-07-26

**Authors:** Marvin Ritter, Olaposi Yomade, Ben-Ole Holtz, Stefanie Deinhardt-Emmer, Aaron Lawson McLean, Stefanie Hartinger, Julia Bechwar, Matthias Schwab, André Huss, Christian Mawrin, Hubertus Axer, Karin G. Schrenk, Philipp A. Reuken, Irina Mäurer

**Affiliations:** ^1^ Department of Neurology, Jena University Hospital, Friedrich Schiller University, Jena, Germany; ^2^ Department of Hematology and Medical Oncology, Clinic of Internal Medicine II, Jena University Hospital, Jena, Germany; ^3^ Comprehensive Cancer Center Central Germany (CCCG), Jena, Germany; ^4^ Institute of Medical Microbiology, Jena University Hospital, Friedrich Schiller University, Jena, Germany; ^5^ Department of Neurosurgery, Jena University Hospital, Friedrich Schiller University, Jena, Germany; ^6^ Department of Neurology, University Hospital of Ulm, Ulm, Germany; ^7^ Department of Neuropathology, Otto von Guericke University Magdeburg, Magdeburg, Germany; ^8^ Department of Pathology, Jena University Hospital, Friedrich Schiller University, Jena, Germany; ^9^ Department of Gastroenterology, Hepatology, and Infectious Diseases, Clinic of Internal Medicine IV, Jena University Hospital, Jena, Germany

**Keywords:** HEV, cancer, lymphoma, neuroinflammation, neurofilaments, neurological symptoms

## Abstract

**Background:**

The hepatitis E virus (HEV) can cause acute viral hepatitis with or without neurological manifestations, and occasionally progresses to chronic infection in immunocompromised individuals. The management of chronic HEV infection in cancer patients may be challenging due to the complex immunological constellation. Furthermore, the diagnostic workflow and the impact on quality of life of neurological HEV manifestations in immunocompromised patients have not been sufficiently delineated previously.

**Case description:**

A 61-year-old male with systemically treated chronic lymphocytic leukemia (CLL) experienced a slowly progressive atrophy of the spinal cord due to a chronic HEV infection. Despite continuous antiviral treatment with ribavirin, the patient’s neurological condition continued to deteriorate, particularly following subsequent attempts to treat CLL. Treatment with obinutuzumab resulted in acute bowel and urinary retention and a further deterioration of motor skills, prompting the discontinuation of obinutuzumab. The patient’s neurological status improved after the administration of intravenous immunoglobulins.

**Conclusion:**

This case study provides a comprehensive long-term follow-up of a cancer patient with chronic HEV infection and associated CNS involvement, which resulted in progressive neurological disability over several years. The challenges faced in diagnosing new neurological symptoms in patients undergoing immunosuppressive cancer treatment underscore the need for an interdisciplinary diagnostic approach that includes HEV testing. We propose a diagnostic pathway for future validation in immunocompromised cohorts presenting with neurological symptoms, emphasizing its potential to enhance clinical outcomes.

## Introduction

Hepatitis E virus (HEV) infection is the primary cause of acute viral hepatitis and typically does not require treatment in immunocompetent patients due to its self-limiting nature (1, 2). However, immunocompromised individuals, such as cancer patients, may develop hepatic fibrosis or cirrhosis due to chronic infection (3, 4). Chronic HEV infection is defined as HEV RNA replication for at least 3 months after the onset of infection (5). Therapeutic options for chronically HEV infected patients include reducing immunosuppressive agents and administering virostatic agents such as ribavirin or pegylated interferon-alpha (4, 5). The HEV seroprevalence, which indicates exposure to HEV through the presence of immunoglobulin G (IgG) antibodies, varies widely among cancer patients (2-63%) in different studies. However, it appears to be at least as high as the seroprevalence observed in the general population (3, 6).

Current data indicates that hematological disorders markedly increase the risk of developing chronic HEV infections. To this extent, a retrospective, multicenter study described a cohort of 50 patients both with hematological malignancies and RNA-positive HEV infection across 11 European centers (3). The authors detected development of chronic hepatitis E in 34% of patients. Ongoing or prior oncological anti-CD20 treatment emerged as the only independent risk factor for HEV chronification. While the mortality linked to the primary hematologic disease significantly surpassed that attributed to the HEV infection, adjustments to oncologic treatment were necessary in the majority of systemically treated cancer patients. These modifications potentially had a significant impact on oncologic outcome, as demonstrated by instances of disease progression and cancer-related deaths (7).

HEV-induced neurologic symptoms are likely to occur in cancer patients due to the relatively high incidence of neurologic manifestations in HEV infections in general (5.5-31%) (8–11). It is uncertain whether the neurological symptoms of HEV are caused by the virus itself or the associated parainfectious immunoinflammation (11). As ribavirin demonstrates limited effectiveness in traversing the blood-brain barrier, an efficacious antiviral treatment for neurological manifestations associated with HEV seems lacking (11–14). Moreover, the exact incidence and consequence of neurological HEV manifestations on the overall quality of life and prognosis in cancer patients is uncertain.

**Figure 1:**
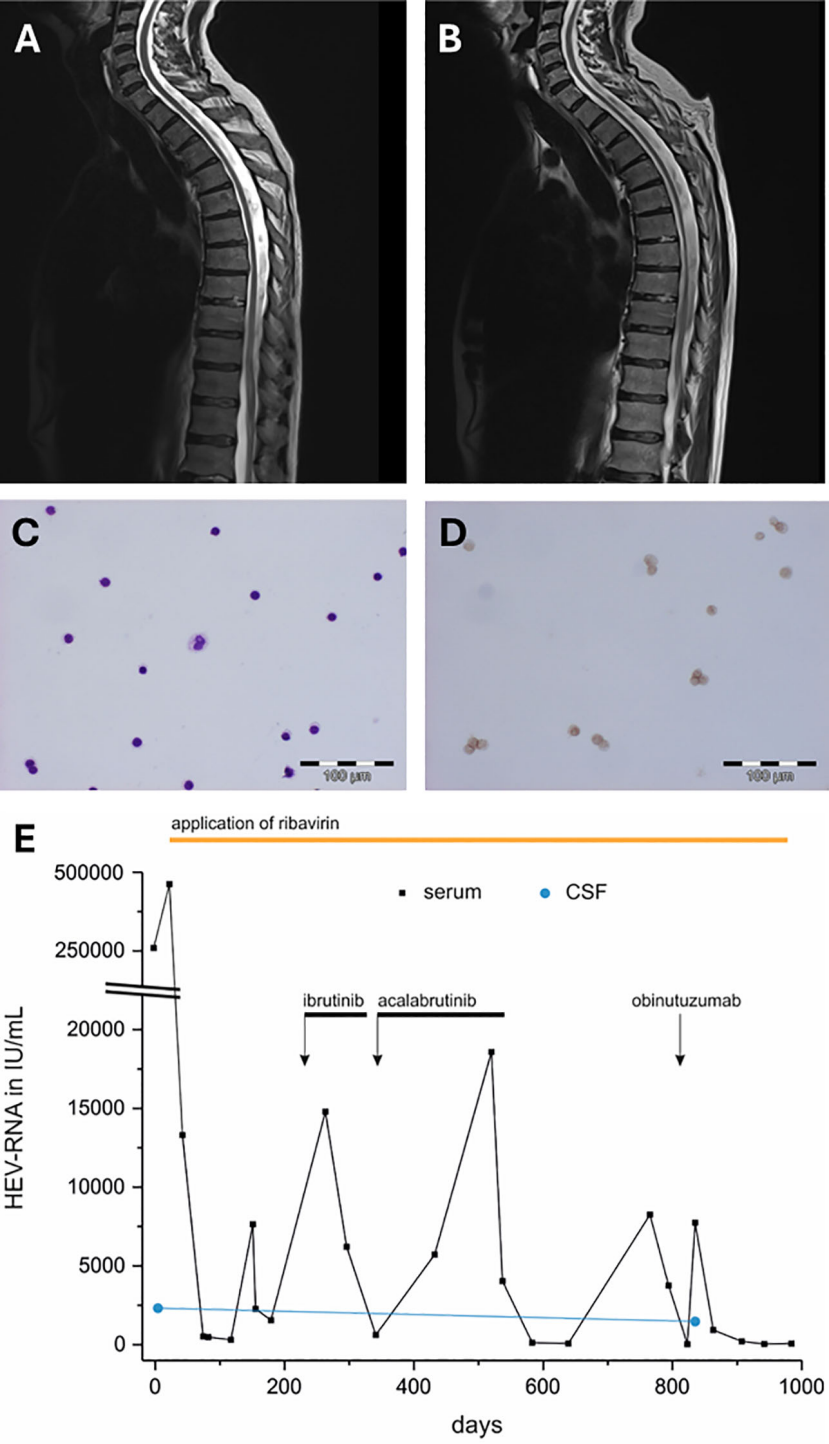
**(A)** MRI (T2 sequence) of the spinal cord in the sagittal plane at the onset of neurological symptoms **(B)** and the progressive spinal cord atrophy after 2 years, directly after the initiation of obinutuzumab. The initial CSF cytology including **(C)** Pappenheim staining, magnification 1:200 and **(D)** CD3 staining of the lymphocytes (magnification 1:200) showed a lymphocytic pleocytosis without evidence of neoplastic infiltration. **(E)** Longitudinal changes in HEV RNA levels following the administration of ibrutinib, acalabrutinib and obinutuzumab (the black arrows represent the start and the black bars the duration of application). The black dots represent HEV PCR measurement timepoints in serum, the blue dots show HEV RNA levels in CSF. The period of application of ribavirin is illustrated with an orange bar. cd, cluster of differentiation; CSF, cerebrospinal fluid; HEV, hepatitis E virus; MRI, magnetic resonance imaging; PCR, polymerase chain reaction; RNA, ribonucleic acid.

We present the long-term observation of a patient suffering from chronic lymphocytic leukemia (CLL) who developed the previously undescribed syndrome of slowly progressing spinal cord atrophy due to chronic HEV infection. Additionally, the patient experienced increased viral replication and acute neurological deterioration after initiation of therapy with the anti-CD20 antibody obinutuzumab. Relevant patient characteristics from the multimodal longitudinal assessment are shown in **Figure 1**, **Table 1**.

**Table 1:** Laboratory results of the patient.

	Initial visit (IV)	6 weeks post IV	3 months post IV	30 months post IV (prior to obinutuzumab)	30 months post IV (after obinutuzumab)	Reference	Unit
**HEV-RNA (plasma)**			267850	37	7746	110	IU/mL
**ASAT**	1.07	–	0.97	–	0.31	0.85	µmol/L*s
**ALAT**	0.94	1.78	1.8	–	0.49	0.83	µmol/L*s
**gGT**	2.67	2.21	2.38	–	1.02	0.17-0.19	µmol/L*s
**HEV-RNA (CSF)**	–	–	2321	–	1478	<110	IU/mL
**Cell count (CSF)**	23	7	17	–	22	<5	cells/µl
**Protein (CSF)**	960	1047	856	–	951	150-400	mg/L
**Lactate (CSF)**	2.8	2.8	2.8	–	2	1.2-2.1	mmol/L
**Glucose-Index (CSF)**	36.8	39,7	46.2	–	45.5	>50	%
**p-Nf-H (CSF)**	8845	5497	–	–	708	62-553	pg/mL
**Nf-L** **(serum)**	83	123	–	–	41	<45	pg/mL

ALAT alanin-aminotransferase; ASAT aspartat-aminotransferase; CSF cerebrospinal fluid; gGT gamma-glutamyl transferase; HEV Hepatitis E virus; IV initial visit; Nf-L light chain neurofilaments; p-Nf-H phosphorylated heavy chain neurofilaments; RNA ribonucleic acid; - not measured.

## Case presentation

A 61-year-old male with a 12-year history of CLL (Binet stage B, no cytogenetic aberrations, Zeta-chain-associated protein kinase (ZAP-70) overexpression) and multiple prior treatment regimens (Fludarabine-cyclophosphamide-rituximab (FC-R) protocol, ibrutinib and idealisib) presented with worsening malaise and abdominal pain following the re-initiation of ibrutinib, a Bruton tyrosine kinase inhibitor (BTKI), given for maintenance therapy of CLL. Shortly thereafter, symmetric paresthesia occurred, initially affecting the feet and fingertips then progressing to above the knees over the course of several months. Neurological clinical assessment revealed distal symmetric hypesthesia of the legs, pallhypesthesia of both medial malleoli, normal tone and brisk lower limb tendon reflexes without plantar extension and reduced lower limb strength with atrophy of small muscle groups leading to unspecific gait disturbance.

While nerve conduction studies showed no evidence of peripheral neuropathy, electromyography demonstrated acute denervation of the left tibialis anterior muscle and chronic denervation of multiple lower limb muscles. Evoked potentials revealed central motor and sensory conduction abnormalities. Examination of cerebrospinal fluid (CSF) showed elevated protein (960 mg/L), cell count (23 cells/µL) and lactate (2.8 mmol/L) without evidence of cancer cells (**Figures 1C, D**). Oligoclonal bands showed no indication of intrathecal antibody production. Analysis of neurofilaments (Nf) revealed considerably increased phosphorylated heavy chain Nf (pNf-H) in CSF 8845 pg/mL (normal range: 62-553 pg/mL) and moderately increased light chain Nf (Nf-L) in the serum 83 pg/mL (normal range: <45 pg/mL). Several blood tests for infectious (e.g. human immunodeficiency virus, lues, mycobacteria, borrelia and neurotropic viruses) and autoimmune conditions remained negative. Suspecting a paraneoplastic or ibrutinib-associated inflammatory syndrome of the central nervous system (CNS), 1000 mg/d methylprednisolone were initially administered for five consecutive days without benefit.

The patient’s neurological condition progressively deteriorated over the next 3 months. Clinical examination revealed progressive loss of strength especially in the upper limbs (left 2/5, right 3/5), brisk tendon reflexes with plantar extension and progressive sensory deficits to all modalities up to a level of T5. Liver enzymes (g-GT 2.38 µmol/L*s, ALAT 1.80 µmol/L*s) were elevated. HEV infection was identified through positive serum HEV RNA (267850 IU/mL). Ultrasound elastography examination with FibroScan^®^ showed hepatomegaly (16cm midclavicular) with stage 4 liver fibrosis (F4). Magnetic resonance imaging (MRI) of the brain and spine showed slight cervical myelopathy of the posterior funiculus of the cervical region (C2-C5; **Figure 1A**). Suspecting funicular myelosis due to slightly decreased folic acid and/or parainfectious myelitis caused by HEV, folic acid was substituted, and intravenous immunoglobulins were administered for 3 days (40 g/d). Ribavirin therapy was initiated (800 mg/d; **Figure 1E**).

Patient follow-up was conducted three months later. Lower limb strength improved slightly, whereas sensory deficits persisted. MRI showed no signs of cervical myelopathy. Polymerase chain reaction (PCR) results revealed high HEV RNA presence in the CSF (2321 IU/mL, cut-off 110 IU/mL).

Over the next 12 months, multiple attempts to adjust immunosuppressive oncologic therapy including the use of ibrutinib or acalabrutinib for CLL due to significant splenomegaly resulted in recurrent episodes of transient increases in HEV RNA (**Figure 1E**) and further neurologic deterioration despite continuation of antiviral therapy with ribavirin.

Two years after the initial diagnosis of the chronic HEV infection, the patient received obinutuzumab for CLL treatment (**Figure 1E**), a humanized anti-CD20 antibody, at a dose of 1000 mg/d. He subsequently developed bowel and urinary retention. Neurological symptoms included reduced sphincter tone without anogenital paresthesia as well as worsened motor weakness of the lower limbs. MRI of the spine revealed increasing atrophy of the thoracic spinal cord (**Figure 1B**). Concurrently measured liver enzymes were within the normal range. PCR of serum HEV demonstrated a significant increase from 37 IU/mL (last measurement prior to CD20 blockade) to 7746 IU/mL while the results of the CSF examination were consistent with the initial neurological consult, showing an elevated cell count of 22 cells/mL, a protein level of 951 mg/dL, and increased HEV level of 1478 IU/mL (initial CSF HEV RNA level 2 years earlier: 2321 IU/mL). Nf in serum were normal (41 pg/mL), Nf in CSF were slightly increased (708 pg/mL). No other infectious, autoimmune, or paraneoplastic etiologies were identified as potential contributors to the worsening of symptoms. Obinutuzumab was immediately discontinued, and intravenous immunoglobulins were administered for five consecutive days at a dose of 30 g/d. Bowel and urinary retention gradually improved, but residual motor deficits persisted compared to the patient’s neurological status before receiving obinutuzumab.

## Interdisciplinary management algorithm proposal

**Figure 2:**
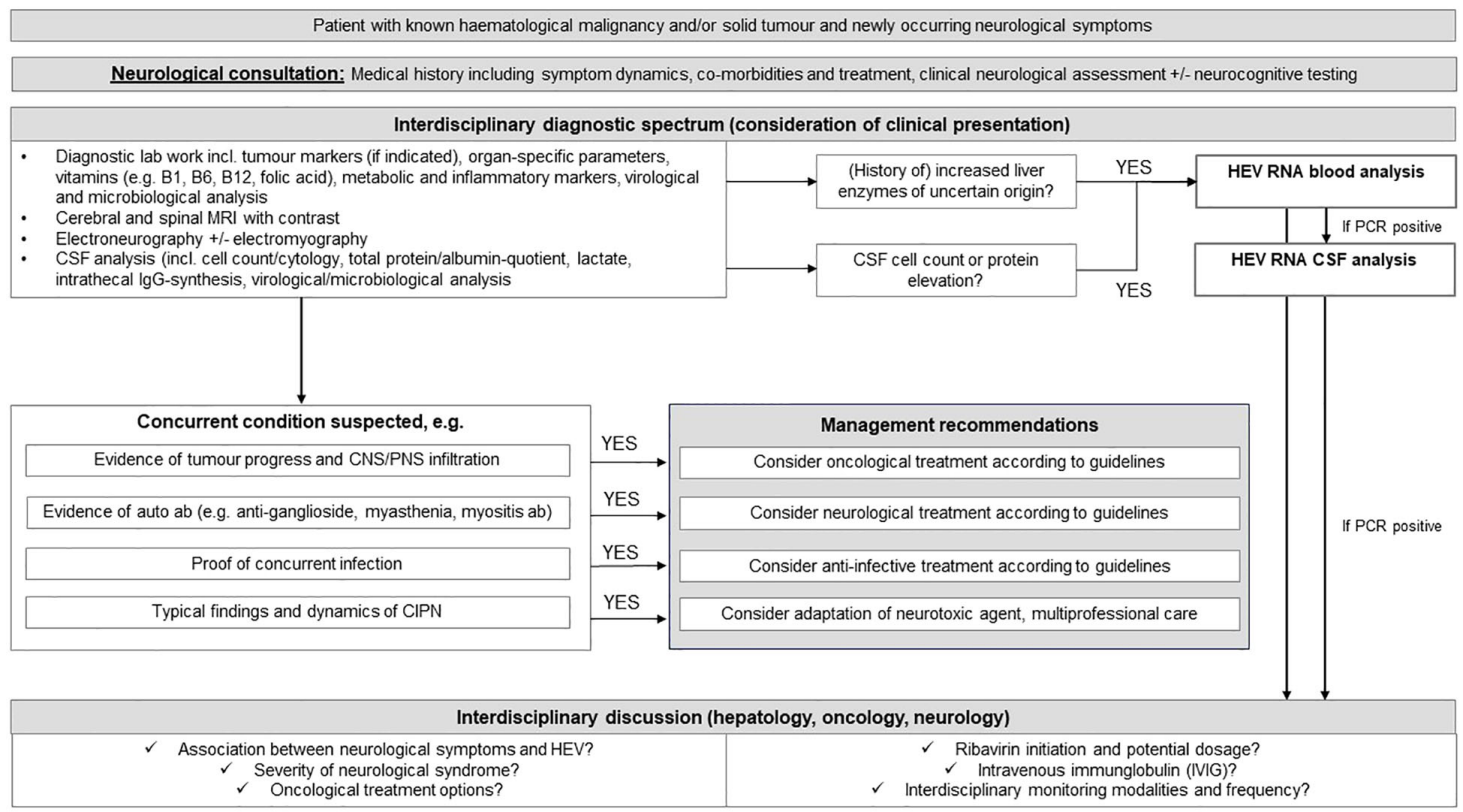
The figure displays the suggested treatment algorithm for the interdisciplinary management of immunocompromised cancer patients with neurological symptoms and an atypical constellation considering the critical aspects of HEV infection. MRI, magnetic resonance imaging; CSF, cerebrospinal fluid; IgG, Immunoglobulin G; CNS, central nervous system; PNS, peripheral nervous system; CIPN, chemotherapy-induced polyneuropathy; HEV, hepatitis E virus; PNS, peripheral nervous system; RNA, ribonucleic acid.

To facilitate decision making in immunocompromised cancer patients, we developed a comprehensive cross-sectional diagnostic pathway that incorporates HEV screening (**Figure 2**). By considering early HEV testing in patients presenting unclear or atypical neurological syndromes and/or inflammation, our management algorithm may mitigate misinterpretations and prevent avoidable long-term risks in this vulnerable patient cohort.

## Discussion

To the best of our knowledge, chronic spinal cord atrophy with chronic HEV infection and persistent HEV RNA in the CSF has not been previously described in the published literature. In this case, the patient was diagnosed with HEV during immunosuppressive ibrutinib treatment. The persistence of HEV despite antiviral treatment with ribavirin led to CNS involvement, manifesting as posterior myelopathy and thoracic spinal cord atrophy during chronic HEV infection. This is evidenced by the consistently abnormal cell count and the recurrent detection of HEV RNA in the CSF, along with gradual progression of the disease. Studies indicate that HEV infection causes both hepatic and extrahepatic manifestations, with neurological symptoms being the most common among extrahepatic effects. Both acute and chronic HEV can cause extrahepatic manifestations, though these are more common in acute infections (15). While the underlying pathomechanism remains unclear, neurotropic affection with viral replication in the CNS and autoimmune reactions due to molecular mimicry and neurotropic affection with viral replication in the CNS have been previously discussed (12, 13, 16). *In vitro* and *in vivo* studies show that HEV infects microvascular endothelial cells, crosses the blood-brain barrier and can subsequently infect the CNS (17). Furthermore, Drave et al. demonstrated that HEV can replicate in human nerve cells, with neurological manifestations being primarily triggered by genotype 3 and to a lesser extent by genotype 1 (18).

Increased Nf levels in the CSF and blood are indicators of neuroaxonal injury and are used to monitor disease severity in various neurological disorders including amyotrophic lateral sclerosis, Parkinson disease, and the neurotoxicity of immune checkpoint inhibitors (19, 20). This case describes elevated serum and CSF Nf levels in a patient with chronic HEV infection and neurological symptoms. Nf could potentially serve as an indicator of neuronal damage severity in HEV-infected patients. However, due to the limited specificity of Nf, it is necessary to rule out non-HEV causes of elevated Nf levels and to acquire baseline measurements before initiating immunosuppressive therapy.

Studies have shown that indolent non-Hodgkin lymphoma, anti-CD20 treatment and rituximab-based chemotherapy are risk factors for HEV infection chronicity (3). In this case, acute neurological deterioration, presenting with a clinical picture of transverse myelitis, occurred following the initiation of immunosuppressive therapy with the anti-CD20 antibody obinutuzumab. This study presents the first report of an increase in HEV replication in the context of neurological deterioration of a cancer patient following the administration of obinutuzumab. While cell count and the protein levels of inflammation markers in the CSF remained stable over several years, the serum HEV PCR level increased, coinciding with changes in neurological deterioration. Our case supports the notion that neurological deterioration may be attributed to the immunosuppression-induced amplification of the HEV itself, rather than solely to the concomitant inflammatory processes.

A large multicenter retrospective study which demonstrated eradication of HEV in 80% of patients treated with ribavirin suggested that early initiation of treatment seems to be beneficial and shortens viremia in immunocompromised patients (3, 21). While the majority of the literature indicates that HEV infections in immunocompromised patients are as prevalent as in the general population, individuals with underlying hematologic malignancies or undergoing anti-CD20 treatment face a significantly higher risk of developing chronic HEV infection (3, 6). The occurrence of HEV infection during ibrutinib therapy in patients with CLL has been previously described. Additionally, Protin et al. observed that ibrutinib-associated HEV-infected patients undergoing ribavirin therapy exhibited a poor response, resulting in chronic infection in a significant proportion of affected individuals (22). This limited response may be attributable to the immunosuppressive effect of ibrutinib itself, as well as to the upregulation of Bruton’s tyrosine kinase during HEV replication (23). Another explanation for the persistence of HEV despite consistent administration of ribavirin is the emergence of in-host HEV quasispecies. Colomer-Castell et al. suggest that mutagenic antivirals like ribavirin, when used in the presence of high viral loads, can lead to the selection and proliferation of a diverse set of synonymous haplotypes expressing the same phenotype. This promotes the selection and proliferation of conservative substitutions that express fitness-enhanced phenotypes. Ribavirin monotherapy can therefore result in highly diversified quasispecies that are resistant to further therapy, which has significant clinical implications (24).

The differentiation of various neurological syndromes in cancer patients, including autoimmune processes, neurotoxicity from systemic treatment, CNS infection, or tumour progression with infiltration of the nervous system, poses a challenge for the treating physician and demands highly interdisciplinary patient management. As mentioned previously, the progressive worsening of the neurological condition of our patient was initially interpreted as a therapy-related neuropathy caused by ibrutinib. This may lead to HEV infected cancer patients being treated with immunosuppressive drugs such as steroids or rituximab due to a primary suspicion of autoimmune or paraneoplastic etiology of the neurological symptoms. The fact that HEV PCR testing is not routinely included in neurological algorithms partially explains the issue. Hence, it is crucial to raise awareness of HEV and its potential harm to oncological and neurological outcomes.

Overall, this case demonstrates the difficulty of managing chronic HEV infections in cancer patients with neurological symptoms. Reduction of immunosuppressive treatment such as targeted therapy or cytostatic agents is often not feasible as it may be associated with tumour progression and reduced overall survival (7, 21). Simultaneously, HEV treatment delays may also result in increased mortality. If ribavirin treatment is not effective in eradicating the virus, treatment options are limited. It is important to emphasize that the significance of managing HEV infections and their impact on the overall outcomes of cancer patients, with or without neurological manifestations, has not yet been sufficiently delineated. Attending physicians should be aware of these complexities and the necessity for the involvement of multidisciplinary teams.

## Conclusion

Modern oncology employs multimodal treatment strategies, integrating various immunomodulatory therapies. However, distinguishing between autoimmune, infectious, and tumour-related neurological disorders and finding the appropriate therapeutic regimen pose significant challenges in cancer patients undergoing immunosuppressive treatment. We propose an interdisciplinary diagnostic pathway for cancer patients experiencing neurological deterioration, aiming to detect HEV early while ruling out concurrent causal factors. In this context, the utility of Nf as diagnostic marker and longitudinal follow-up parameter for neural damage accompanying HEV manifestations warrants validation in HEV cohorts with and without neurological symptoms.

## Data availability statement

The raw data supporting the conclusions of this article will be made available by the authors, without undue reservation.

## Ethics statement

The studies involving humans were approved by Ethics Committee of Medical Faculty Jena (2024-3319-Daten, approved on the 8th of March 2024). The studies were conducted in accordance with the local legislation and institutional requirements. The participants provided their written informed consent to participate in this study. Written informed consent was obtained from the individual(s), and minor(s)’ legal guardian/next of kin, for the publication of any potentially identifiable images or data included in this article.

## Author contributions

MR: Conceptualization, Data curation, Formal Analysis, Investigation, Methodology, Project administration, Validation, Visualization, Writing – original draft, Writing – review & editing. OY: Data curation, Investigation, Supervision, Validation, Writing – review & editing, Resources. BH: Data curation, Investigation, Validation, Visualization, Writing – review & editing. SD: Data curation, Investigation, Resources, Supervision, Validation, Writing – review & editing. AM: Data curation, Investigation, Validation, Writing – review & editing. SH: Data curation, Investigation, Project administration, Validation, Writing – review & editing. JB: Validation, Writing – review & editing. MS: Conceptualization, Data curation, Investigation, Resources, Supervision, Validation, Writing – review & editing. AH: Investigation, Writing – review & editing. CM: Investigation, Visualization, Writing – review & editing. HA: Investigation, Resources, Supervision, Validation, Writing – review & editing. KS: Data curation, Investigation, Resources, Supervision, Validation, Writing – review & editing. PR: Conceptualization, Data curation, Investigation, Methodology, Project administration, Resources, Supervision, Validation, Visualization, Writing – review & editing. IM: Conceptualization, Data curation, Formal Analysis, Funding acquisition, Investigation, Methodology, Project administration, Software, Supervision, Validation, Visualization, Writing – original draft, Writing – review & editing.
